# Visual Impairment Is Associated With Depressive Symptoms—Results From the Nationwide German DEGS1 Study

**DOI:** 10.3389/fpsyt.2018.00114

**Published:** 2018-04-09

**Authors:** Alexander K. Schuster, Jonas Tesarz, Jasmin Rezapour, Manfred E. Beutel, Bernd Bertram, Norbert Pfeiffer

**Affiliations:** ^1^Department of Ophthalmology, University Medical Center Mainz, Mainz, Germany; ^2^Department of General Internal Medicine and Psychosomatics, Medical Hospital, University of Heidelberg, Heidelberg, Germany; ^3^Department of Psychosomatic Medicine and Psychotherapy, University Medical Center Mainz, Mainz, Germany; ^4^Private Practice for Ophthalmology, Aachen, Germany

**Keywords:** epidemiology, visual impairment, depressive symptoms, Patient Health Questionnaire-9, reading, face recognition

## Abstract

**Introduction:**

Visual impairment (VI) is associated with a variety of comorbidities including physical and mental health in industrial countries. Our aim is to examine associations between self-reported impairment and depressive symptoms in the German population.

**Methods:**

The point prevalence of self-reported VI in Germany was computed using data from the German Health Interview and Examination Survey for adults from 2008 to 2011 (*N* = 7.783, 50.5% female, age range 18–79 years). VI was surveyed by two questions, one for seeing faces at a distance of 4 m and one for reading newspapers. Depressive symptoms were evaluated with the Patient Health Questionnaire-9 questionnaire and 2-week prevalence was computed with weighted data. Depressive symptoms were defined by a value of ≥10. Logistic regression analysis was performed to analyze an association between self-reported VI and depressive symptoms. Multivariable analysis including adjustment for age, gender, socioeconomic status, and chronic diseases were carried out with weighted data.

**Results:**

The 2-week prevalence of depressive symptoms was 20.8% (95% CI: 16.6–25.7%) for some difficulties in distance vision and 14.4% (95% CI: 7.5–25.9%) for severe difficulties in distance vision, while 17.0% (95% CI: 13.3–21.4%), respectively, 16.7% (95% CI: 10.7–25.1%) for near vision. Analysis revealed that depressive symptoms were associated with self-reported VI for reading, respectively, with low VI for distance vision. Multivariable regression analysis including potential confounders confirmed these findings.

**Conclusion:**

Depressive symptoms are a frequent finding in subjects with difficulties in distance and near vision with a prevalence of up to 24%. Depressive comorbidity should therefore be evaluated in subjects reporting VI.

## Introduction

Visual impairment (VI) is associated with a variety of comorbidities including physical health (1), mental health (2), poverty, and social deprivation (3) in industrial countries. The main reasons for moderate and severe vision impairment in high-income countries are uncorrected refractive error, cataract, macular degeneration, glaucoma, and diabetic retinopathy (4). Nowadays, there are treatment options for the majority of these diseases, nevertheless there is still a sizeable number of subjects with moderate and severe VI (presenting visual acuity <6/18, but ≥3/60 in the better eye), namely, 1.0% in high-income countries (Central Europe, Western Europe, North America, Australasia, Pacific Asia) in 2010 (point prevalence) (4). Decreased vision does not only reduce mobility but also limits activity of daily life and restricts social participation (5); similar impairments are also seen in subjects with depression.

A recent systematic review showed that the prevalence of depression or depressive symptoms is 25% among eye disease patients, with similar prevalence for different eye diseases namely dry eye disease (29%), glaucoma (25%), age-related macular degeneration (24%), and cataract (23%) (6). The authors summarized findings from 1-week prevalence data mainly. Accordingly, a person with an eye disease is 1.6 times more likely to suffer from depression compared to a healthy control (6). With respect to eye diseases, Eramudugolla et al. investigated Australians with age-related eye diseases and found a prevalence of 6.7% in cataract patients, 4.5% in glaucoma patients, and 10.5% in those with age-related macular degeneration in the general population using the Goldberg scales (7) and Wang et al. found a 2-week prevalence of depression of 10.9% in subject with glaucoma in the US (8).

While many studies investigating VI and depressive symptoms focused on clinical samples, some studies investigated the association of self-reported VI with depressive symptoms in the general population (1, 9). Garin et al. reported an association between subjective visual acuity and major depression within the last 12 months in a Spanish cohort over the age of 18 years (1, 9), while Zhang et al. found an association between self-reported visual function and depressive symptoms within the last 2 weeks in 10,480 US adults 20 years of age or older (1, 9).

Moreover, to the best of our knowledge, there are no data available for the German population. Thus, we aimed to investigate the association of self-reported VI with depressive symptoms in a large representative population-based cross-sectional sample of adults living in Germany.

## Materials and Methods

### Study Design

The German Health Interview and Examination Survey for Adults DEGS1 is conducted by the Robert Koch Institute (RKI) as a cohort study (10–12). The aim of DEGS is to collect health data of adults aged between 18 and 79 years. In the baseline survey performed between 2008 and 2011, 8,152 adults from 180 German cities and municipalities were investigated. 3,959 persons had already participated in the German National Health Interview and Examination Survey in 1998 while 4,193 additional persons were recruited for DEGS1 based on two-stage stratified random sampling from local population registries. The study protocol was consented with the Federal and State Commissioners for Data Protection and approved by the Charité-Universitätsmedizin Berlin ethics committee (No. EA2/047/08), and participants provided written informed consent (10–12). The resulting study sample was weighted to be representative for the Federal Republic of Germany (11).

For the present analysis, the DEGS1 Public Use File (13) was used. In the DEGS1 2008–2011 baseline survey, VI and chronic diseases, gender and age, socioeconomic status (SES) (14), and other social and health characteristics were investigated by applying questionnaires. Chronic diseases were surveyed by the following questions: “Do you have one or several long-lasting chronic diseases?” Chronic diseases are long-lasting diseases that need permanent treatment or checkup, i.e., diabetes or cardiac diseases—possible answers were “Yes—No—Do not know.” The answer “Do not know” was set as missing. Age was grouped in 5-year intervals. SES was computed based on education, income, and occupation with a score range from 3 to 21 (14). Categorization into “low” (first quintile: score 3.0–7.7), “medium” (second to fourth quintiles: 7.8–14.1), and “high” (fifth quintile: 14.2–21.0) was performed (14).

### Assessment of VI

Visual impairment was surveyed by the following questions for distance vision: “Can you see the face of a person at a 4-meter-distance (on the other side of the street)? (if necessary with glasses)?” Function of near vision was questioned with: “Can you read the printing of newspapers? (if necessary with glasses).” The answer categories were “yes, without difficulties; yes, with some difficulties; yes, with severe difficulties; no, not at all.” Low VI was defined as “yes, with some difficulties,” moderate to severe VI as “yes, with severe difficulties” and “no, not at all.” This definition was chosen due to the low prevalence of the answers: “yes, with severe difficulties” and “no, not at all.” Therefore, VI was coded as a three-category variable (no VI, low VI, and moderate to severe VI).

### Assessment of Depressive Symptoms

Depressive symptoms were surveyed using the German version of the “Patient Health Questionnaire” (PHQ-9) (15). The PHQ-9 is reported to be a reliable and valid self-assessment instrument for measuring the frequency of being bothered by nine depressive symptoms within the last 2 weeks in community-based setups (16, 17) with a score range from 0 to 27. Items are based on the diagnostic criteria for “major depression” as defined in DSM-IV (little interest or pleasure, depressed mood, sleep disturbances, tiredness, poor appetite or overeating, feelings of worthlessness or guilt, trouble concentrating, psychomotor retardation or agitation, suicidal thoughts) (18). A cutoff of ≥10 was applied for defining depressive symptoms. This cutoff is described to have a pooled sensitivity of 77% and pooled specificity of 85% for any depressive disorder over 13 studies (19).

### Statistical Analysis

Descriptive statistics such as numbers and percentages and means and SEs were used for demographic and clinical variables. Point prevalence estimates for VI (distance vision respectively near vision) and 2-week prevalence estimates were computed.

Logistic regression analysis was used to evaluate the association between depressive symptoms and self-reported VI for distance vision and for reading. Effect estimates [odds ratio (OR)] and their SEs were computed unadjusted (model #1) and adjusted for the following confounders: model #2: age (as categorical variable in 5-year intervals) and gender, SES (categorical variable: low, medium, high), and presence of chronic diseases (dichotomous); and model #3 additionally adjusted for antidepressive medication (dichotomous). Stepwise inclusion of independent variables was not conducted; all variables of the models #2 and #3 were included at once.

Socioeconomic status was coded 1 for “low,” 2 for “medium,” and 3 for “high” with the high category as reference, Age as 1 for “18–24 years,” 2 for “25–29 years,” 3 for “30–34 years,” 4 for “35–39 years,” 5 for “40–44 years,” 6 for “45–49 years,” 7 for “50–54 years,” 8 for “55–59 years,” 9 for “60–64 years,” 10 for “65–69 years,” 11 for “70–74 years,” and 12 for “75–79 years” with the “75–79 years” category as reference. For the dichotomous variables, gender was coded as 1 for male and 2 for female with female as reference category, presence of chronic diseases was coded 1 for “yes” and 2 for “no” with “no” as reference category, and antidepressive medication was coded 1 for “yes” and 2 for “no” with “no” as reference category.

To evaluate the quality of our model, goodness-of-fit statistics were calculated using Nagelkerke’s R^2^, the Hosmer–Lemeshow statistics, and classification table (percentage of correct classification for total study cohort, controls, and subjects with depressive symptoms).

In addition, insufficiently treated depressive subjects [antidepressive medication (ATC-code N06A) = “yes” and PHQ-9 ≥10], sufficiently treated depressive subjects (antidepressive medication = “yes” and PHQ-9 <10), depressive subjects without medication (antidepressive medication = “no” and PHQ-9 ≥10), and normal subjects (antidepressive medication = “no” and PHQ-9 <10) were compared.

The Spearman correlation between the two types of VI was determined to investigate collinearity.

All analyses were carried out using the IBM Statistical Package for the Social Sciences version 24.0 including sampling weights as recommended by the RKI. In addition, we performed a sensitivity analysis and performed logistic regression analysis without sampling weights. Our study is of exploratory nature, variable selection was data driven, and does not attempt to produce causal interference.

## Results

**Figure 1:**
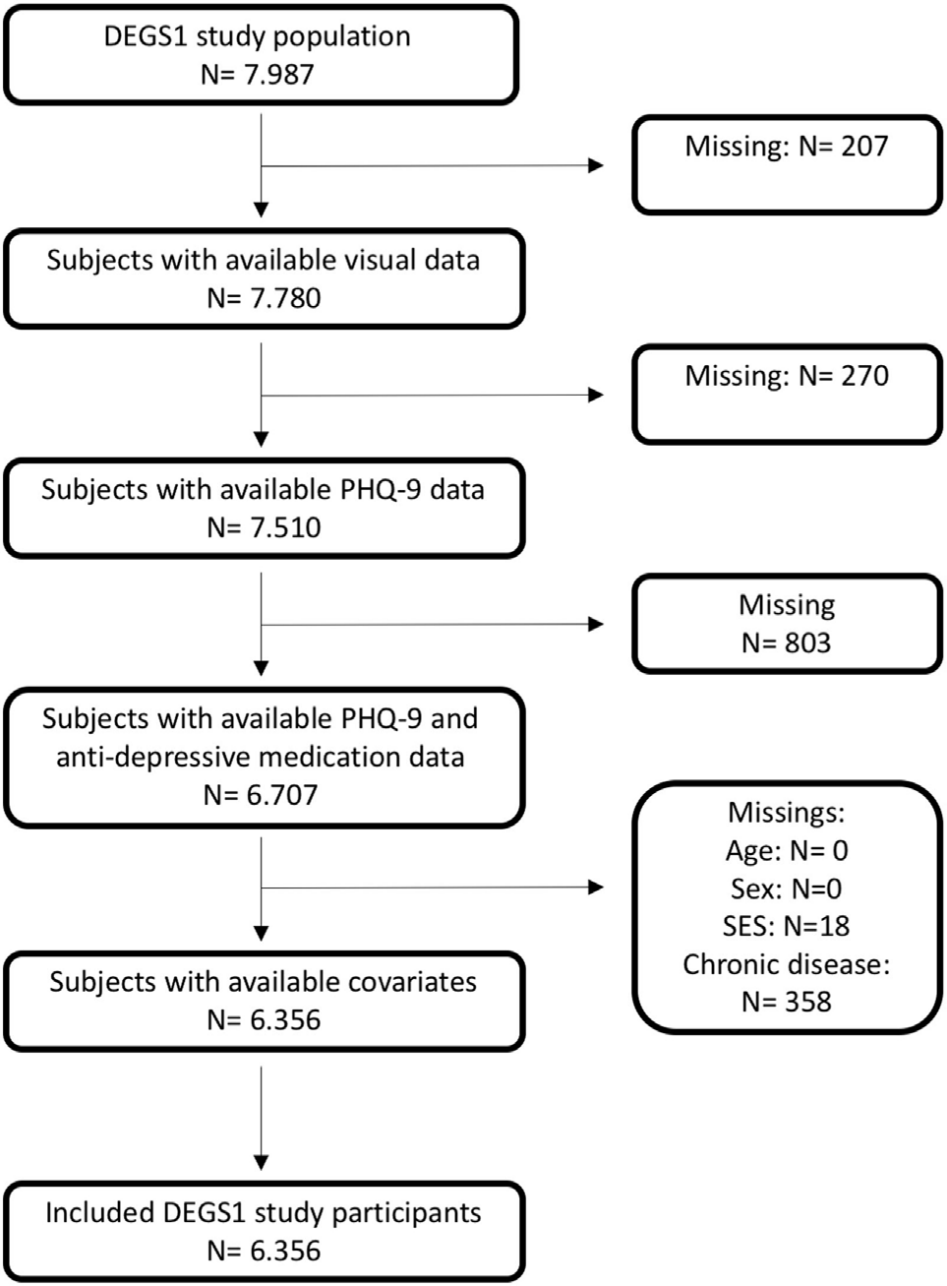
Flow chart of data availability in the DEGS1 study cohort with respect to subjective visual impairment and depressive symptoms.

Of 7.987 subjects between the age of 18 and 79 years, 7.783 (97.4%) subjects had answered the question on VI for both distance vision and reading vision. The characteristics of the total sample and subjects with VI are described in Table 1. Data availability is presented in the flow chart in Figure 1.

**Table 1:** Characteristics of the study sample and the subgroup of subjects with depressive symptoms: results from the DEGS1 study in Germany.

Characteristic (weighted according to the German population)	Total study sample (*N* = 7.780)	Subjects with depressive symptoms (Patient Health Questionnaire-9 ≥10) (*N* = 552)
VI for distance vision (%) • Low • Moderate to severe	7.6 1.5	18.6 2.6

VI for reading (%) • Low • Moderate to severe	9.7 2.3	19.4 4.8

Gender (% female)	50.5	62.6

Age (years) (in percentage of the study population)18–24 25–29 30–34 35–39 40–44 45–49 50–54 55–59 60–64 65–69 70–74 75–79	10.7 8.4 7.1 7.7 10.5 10.9 10.0 8.5 7.2 6.8 8.2 4.0	11.3 12.1 8.4 6.2 10.5 11.6 8.6 10.2 7.0 5.4 5.7 3.1

Residence (rural/small town/town/city) (%)	16.2/23.3/29.5/31.0	14.9/16.6/32.6/35.8

Socioeconomic status (%) Low Medium High	18.9 60.7 20.4	31.3 56.9 11.9

Chronic diseases (yes) (%)^a^	30.3	47.4

Current antidepressive medication (yes) (%)	4.8	18.7

*^a^Chronic diseases were surveyed as long-lasting chronic diseases that need permanent treating or checkups*.

### Point Prevalence for VI at Distance

In brief, 9.1% (95% CI: 8.3–9.9%) of the German population reported at least some difficulties for distance vision. 0.5% (95% CI: 0.3–0.7%) were not able to see faces at all at a 4-m distance; 1.0% (95% CI: 0.7–1.4%) had large difficulties and 7.6% (95% CI: 6.8–8.4%) had some difficulties in seeing faces.

#### Gender Effects

With respect to gender, 4.6% (95% CI: 3.8–5.5%) of the male subjects reported some difficulties for distance vision and 0.5% (95% CI: 0.3–1.0%) large difficulties, whereas 10.5% (95% CI: 9.4–11.8%), respectively, 2.4% (95% CI: 1.9%–3.2%) of the female subjects (*p* < 0.001) did so.

### Socioeconomic Status

Socioeconomic status was different in subjects with difficulties in distance vision (*p* < 0.001). High SES was linked to lower prevalence of difficulties compared to medium SES, while low SES showed the highest prevalence.

### Point Prevalence for VI in Reading

For near vision defined as reading newspaper, 12.0% (95% CI: 11.0–13.1%) of the German population had at least some difficulties. 1.0% (95% CI: 0.8–1.4%) reported that reading newspaper was not possible at all; 1.3% (95% CI: 1.0–1.6%) had large difficulties and 9.7% (95% CI: 8.8–10.7%) had some difficulties in doing so.

#### Gender Effects

In contrast to distance vision, there were less gender differences in near vision: 9.3% (95% CI: 8.2–10.6%) of the male subjects reported some difficulties for reading and 1.8% (95% CI: 1.3–2.4%) severe difficulties, while 10.1% (95% CI: 8.7–11.5%), respectively, 2.9% (95% CI: 2.2–3.7%) of the female subjects did so (*p* = 0.04).

#### Socioeconomic Status

Similar to distance vision, SES was different in subjects with VI in reading (*p* < 0.001). High SES was linked to lower prevalence of difficulties compared to medium SES, while low SES had the highest prevalence.

Visual impairment for distance vision and for reading correlated weakly with each other (rho = 0.25, *p* < 0.001), indicating that the two items represent different characteristics of VI.

### 2-Week Prevalence of Depressive Symptoms in Subjects With VI

Depressive symptoms (PHQ-9 score ≥10) were more frequent in subjects with self-reported VI: 20.8% (95% CI: 16.6–25.7%) of subjects with some difficulties in distance vision and 14.4% (95% CI: 7.5–25.9%) with severe visual difficulties for distance; and 17.0% (95% CI: 13.3–21.4%), respectively, 16.7% (95% CI: 10.7–25.1%) for near vision were identified as suffering from depressive symptoms, while this was the case for 8.1% (95% CI: 7.3–9.1%) of the subjects reporting no difficulties in seeing faces, respectively, 7.0% (95% CI: 6.1–7.9%) in reading a newspaper.

### Logistic Regression Results

A logistic regression analysis was conducted to predict depression with age, gender, SES, chronic disease status, and antidepressive drugs as predictors for both weighted data (Table 2) and unweighted data (Table S1 in Supplementary Material). As summary statistics of model fit Nagelkerke’s R-square and percentage of correct classification were calculated. Nagelkerke’s R-square increase from 0.03/0.02 (VI in distance/reading) in the crude analysis to 0.13/0.12 in the adjusted model #2 (Table S1 in Supplementary Material). Percentage of correct classification was approximately 93% with few correct classifications for subjects with depressive symptoms.

**Table 2:** Associations of visual impairment (VI) and depression: results from the DEGS1 study presenting representative data for the German population with an age range from 18 to 79 years (*N* = 6.356).

Depression (Patient Health Questionnaire-9 score ≥10)	Crude odds ratio (OR)	Adjusted OR_M1_	Adjusted OR_M2_
VI in seeing faces at distance Low	3.90 [2.81; 5.43]	3.08 [2.24; 4.24]	3.03 [2.21; 4.14]
Moderate to severe (reference: no)	2.29 [0.98; 5.38]	1.66 [0.68; 4.09]	1.39 [0.52; 3.72]
VI in reading Low	2.69 [1.91; 3.80]	2.47 [1.72; 3.55]	2.36 [1.64; 3.40]
Moderate to severe (reference: no)	2.71 [1.49; 4.96]	2.04 [1.09; 3.80]	2.01 [1.03; 3.92]

*Results are given as crude and adjusted OR [95% confidence intervals] calculated by logistic regression analysis*.

*Model 1 (M1): adjusted for age (in 5-year intervals), gender, socioeconomic status (SES), and presence of chronic disease*.

*Model 2 (M2): adjusted for age (in 5-year intervals), gender, SES, presence of chronic dis ease, and intake of antidepressive drugs*.

*Including only one type of VI (either recognizing faces at distance or reading) in the logistic regression model to test its association with depressive symptoms*.

The logistic regression analyzes revealed that, compared to the reference category no VI, low VI for distance was associated with depressive symptoms (crude OR = 3.90, 95% CI: 3.82–5.43, adjusted OR_M1_ = 3.08, 95% CI: 2.24–4.24, adjusted OR_M2_ = 3.03, 95% CI: 2.21–4.14), while moderate to severe VI was not associated with depressive symptoms in all three models (Table 2). VI in reading was associated with depressive symptoms in univariate analysis (low vs. no VI: OR = 2.69, 95% CI: 1.91–3.80; moderate to severe vs. no VI: OR = 2.71, 95% CI: 1.49–4.96). Adjustment for potential confounders showed lower associations (low vs. no VI: OR_M1_ = 2.47, 95% CI: 1.72–3.55; OR_M2_ = 2.36, 95% CI: 1.64–3.40; moderate to severe vs. no VI: OR_M1_ = 2.04, 95% CI: 1.09–3.80; OR_M2_ = 2.01, 95% CI: 1.03–3.92; Table 2).

Sensitivity analysis applying logistic regression analysis without weighted data showed comparable associations of depressive symptoms with subjective VI (Table S1 in Supplementary Material).

## Discussion

This study analyzes the association of self-reported VI with depressive symptoms in a large representative population-based cross-sectional sample of adults living in Germany between 2008 and 2011. Our results show that (1) subjective VI is a common finding in the German population and (2) people with self-reported VI have an almost threefold higher 2-week prevalence of clinical relevant depressive symptoms. This increase is independent from age, gender, and SES.

The point prevalence of at least some VI in face recognition is 9.1% in the German population and for reading 12.0%. This is comparable to results from a prior investigation in Germany, showing a point prevalence of difficulties in face recognition in 19.5% of the subjects. That study, however, did not discriminate between difficulties in distance and near visual tasks (20). Other countries show similar VI rates: the Nord-Trøndelag Health Study showed a prevalence of self-reported VI in 17.0% in Norwegians (21) having surveyed longstanding impairments of at least 1 year. In the US, a point prevalence of 9.7% of functional VI was reported in 2010. 16 years earlier, in 1984, the point prevalence had been 23.3% (22). With regard to gender, women indicated to experience more problems from VI for distance: 72% of subjects with VI in face recognition were female, while there was no relevant gender difference for VI in reading.

With respect to mental health, our results showed a clear association between VI and depressive symptoms for both distance visual tasks and near visual tasks, independent from age, gender, SES, and presence of chronic diseases. Busch et al. reported that persons of higher SES are less likely to have current depressive symptoms (18). Our data show that persons of higher SES are also less likely to have VI, and we therefore adjusted our analysis for SES. Nevertheless, it needs further investigation to identify cause and consequence of this association. There is a controversy in literature over the relationship between VI and depression as some studies report such an association (1, 23, 24) while others do not (25, 26). The variability between these studies may be caused by different types of visual assessment such as objective visual acuity, subjective visual function, medical record review, and presence of age-related eye disease. Interestingly, Garin et al. and Zhang et al. reported that self-reported visual function loss rather than loss of visual acuity is linked to depression (1, 9) Therefore, Garin et al. analyzed associations with major depression within the last 12 months (1), while Zhang et al. examined depressive symptoms within the last 2 weeks (9). Depressive persons might perceive their vision as worse compared to non-depressive persons, while objective measures such as visual acuity do not show this discrepancy. Nevertheless, self-reported VI and visual acuity measures consider different aspects of vision. While visual acuity measures the aligning discrimination of the eye, visual function tasks integrate more components such as the circuitry of the neuronal network to be able to keep the line when reading and to perform line skips. In addition, self-experienced VI is of importance as this may result in disability, functional loss, and social isolation (26, 27). Interestingly, we did not find a stronger association with respect to degree of VI, but rather the other way round. This may show that rather the experience of VI than its amount is the relevant factor.

We think it is remarkable that the association between VI for distance vision tasks and depressive symptoms was stronger than for near-vision tasks. This is in accordance with a finding of the Spanish Cohort of the COURAGE study which found stronger associations between subjective visual acuity and major depression within the last year than for subjective near visual acuity (1). This indicates that the ability to recognize objectives at distance might have a stronger impact on depression than the ability to read.

Our study has several limitations. First, it did only investigate subjects within an age range from 18 to 79 years. Thus, we are not able to draw any conclusions on the very elderly that are even more affected by VI. The use of a self-report questionnaire to assess depressive symptoms and not having an interview-based diagnostic assessment does not allow to differentiate between subtypes of depression. Depression is a heterogeneous disorder and different subtypes might have been differentially associated with VI. Although depressive symptoms were investigated by the validated PHQ-9 questionnaire, the questionnaire does not allow to differentiate regarding the severity of depressive symptoms, for which a comprehensive examination would be necessary. Moreover, the use of the PHQ-9 only captures a smaller percentage of depressive symptoms compared to direct diagnostic interviews, which may have affected the observed associations.

In addition, goodness of fit in the different statistical models was low and especially in subjects with depressive symptoms the percentage of correct classification was limited. Therefore, our results should be interpreted with caution.

Furthermore, cross-sectional studies do not allow to draw conclusions on cause and effect of relationships, but they merely analyze associations. We were not able to adjust for differences in compliance, for instance depressive subjects might not have the best suiting spectacle correction and therefore experience their vision as being worse. Also, we were not able to incorporate whether best correcting eyeglasses or contact lenses were used by both depressive and non-depressive subjects and differences in usage might explain our results. Knowing whether depression symptoms existed before, while, or after VI occurred, might affect the reported association.

Despite these limitations, the strengths of this study should also be considered. Up to now, data supporting conclusions on the relationship between VI and depressive symptoms in the German population have been unavailable. Closing this gap, our results indicate for the first time that both subjective VI for distance and for near are associated with depressive symptoms. Interestingly, this association was present only for low subjective VI for distance, while moderate to severe subjective VI was not linked to depressive symptoms. VI was not validated by the assessment of visual acuity, and our results have to be replicated by objective measurements yet, but from a patient’s perspective, the subjective experience is at the forefront as endorsed by our results. These may be interpreted that low VI may rather be linked with depressive symptoms than moderate to severe VI. Adjustment for antidepressive therapy did not change our findings and effect estimates (ORs) were comparable. As further limitation, goodness of fit of our statistical models was low, as was correct classification, especially of subjects with depressive symptoms. Therefore, reported associations should be interpreted cautiously.

Nevertheless, there was a difference in VI with respect to treatment status of depressive symptoms. Not sufficiently treated depressive subjects (taking antidepressive medication and having a PHQ-9 score ≥10) had the highest prevalence of at least some VI for face recognition and reading newspaper, followed by untreated depressive subjects, sufficiently treated subjects (PHQ-9 score <10), and subjects without depressive symptoms (PHQ-9 score <10; Table S2 in Supplementary Material). These data suggest that sufficient treatment of depression might go along with less subjective VI.

In conclusion, subjective VI is a common finding in the German population with a point prevalence of 9.1% for distance vision tasks and 12.0% for reading. People with such a VI have an almost threefold higher prevalence of clinical relevant depressive symptoms. This increase is independent from age, gender, and SES. The challenge now is to identify the underlying mechanisms and to investigate whether therapy of depression is linked to lower prevalence of subjective VI, as our epidemiological cross-sectional data suggest.

## Ethics Statement

This study was carried out in accordance with the recommendations of the data protection regulations set out in the German Federal Data Protection Act and was approved by the German Federal Commissioner for Data Protection and Freedom of Information. DEGS1 was approved by the ethics committee of the Charité-Universitätsmedizin Berlin (No. EA2/047/08). Participation in the study was voluntary. The participants were fully informed about the study’s aims and content, and about data protection. All participants provided written informed consent.

## Author Contributions

AS and JT designed the analysis idea; AS performed the statistics; AS, JT, JR, MB, BB, and NP interpreted the results; AS, JT, JR, MB, BB, and NP drafted the manuscript; and AS, JT, JR, MB, BB, and NP finally approved the manuscript.

## Conflict of Interest Statement

The authors declare that the research was conducted in the absence of any commercial or financial relationships that could be construed as a potential conflict of interest.
