# Supplementary material for: Visual Impairment Is Associated With Depressive Symptoms—Results From the Nationwide German DEGS1 Study
Source: Front Psychiatry. 2018 Apr 9;9:114. doi: 10.3389/fpsyt.2018.00114 (PMC5900411; doi:10.3389/fpsyt.2018.00114)
Supplement: Supplementary file 1 [file table_1.docx]

**Supplemental material #1:**

**Sensitivity analysis:** Associations of visual impairment and depressive symptoms without weighted data: results from the DEGS1-study presenting representative data for the German population within an age range from 18 to 79 years (n=6.356)

| **Depression (PHQ-9 score** ≥**10)**  **Model #1** | **Crude OR** | **Modelling (Nagel-kerkes R-square)** | **Hosmer-Lemeshow-Test**  chi-square (p-value) | **Percentage of correct classification**  **Overall (controls/subjects with depressive symptoms)** |
| --- | --- | --- | --- | --- |
| Visual impairment in seeing faces at distance  low  moderate to severe  (reference: no)  Visual impairment in reading  low  moderate to severe  (reference: no) | 3.72 [2.87; 4.81]  2.57 [1.35; 4.91]  2.52 [1.94; 3.26]  2.12 [1.26; 3.57] | 0.03  0.02 | 0.000 (.^1^)  0.000 (.^1^) | 93.1% (100.0% / 0.0%)  93.1% (100.0% / 0.0%) |

^1^ p-value calculation was not able due to small chi-square.

| **Depression (PHQ-9 score** ≥**10)**  **Model #2** | **Adjusted OR^*^** | **Modelling (Nagel-kerkes R-square)** | **Hosmer-Lemeshow-Test**  chi-square (p-value) | **Percentage of correct classification**  **Overall (controls/subjects with depressive symptoms)** |
| --- | --- | --- | --- | --- |
| Visual impairment in seeing faces at distance  low  moderate to severe  (reference: no)  Visual impairment in reading  low  moderate to severe  (reference: no) | 3.17 [2.42; 4.15]  1.90 [0.98; 3.68]  2.37 [1.80; 3.12]  1.76 [1.02; 3.01] | 0.09  0.08 | 9.04 (0.34)  6.23 (0.62) | 93.2% (100.0% / 0.5%)  93.1% (100.0% / 0.0%) |

| **Depression (PHQ-9 score** ≥**10)**  **Model #3** | **Adjusted OR^#^** | **Modelling (Nagel-kerkes R-square)** | **Hosmer-Lemeshow-Test**  chi-square (p-value) | **Percentage of correct classification**  **Overall (controls/subjects with depressive symptoms)** |
| --- | --- | --- | --- | --- |
| Visual impairment in seeing faces at distance  low  moderate to severe  (reference: no)  Visual impairment in reading  low  moderate to severe  (reference: no) | 3.15 [2.39; 4.16]  1.84 [0.94; 3.61]  2.34 [1.77; 3.10]  1.79 [1.02; 3.13] | 0.13  0.12 | 6.90 (0.55)  4.92 (0.77) | 93.0% (99.7% / 2.1%)  93.2% (99.9% / 2.7%) |

Results are given as crude and adjusted odds ratios (OR) [95% confidence intervals] calculated by logistic regression analysis.

* Adjusted for age (in 5-years intervals), gender, socio-economic status and presence of chronic disease

# Adjusted for age (in 5-years intervals), gender, socio-economic status, presence of chronic disease and intake of anti-depressive drugs

Including only one type of visual impairment (either recognizing faces at distance or reading) in the logistic regression model to test its association with depressive symptoms.

**Supplemental material #2:**

Visual impairment in dependence on anti-depressive medication and depressive symptoms: results from the DEGS1-study presenting representative data for the German population with an age range from 18 to 79 years (n=6.707).

| **Prevalence and 95% confidence interval** | **Insufficiently treated depressive subjects** | **Sufficiently treated depressive subjects** | **Untreated depressive subjects** | **Control subjects** |
| --- | --- | --- | --- | --- |
|  | N= 82 | N= 232 | N= 398 | N= 5995 |
| Visual impairment in  recognizing faces at distance  (at least some)  Visual impairment in reading  (at least some) | 30.6% [18.3 – 46.4%]  37.1% [24.8 – 51.3%] | 13.1% [8.6 - 19.5%]  14.9% [9.6 - 22.4%] | 18.9% [14.8 - 23.9%]  20.6% [15.9 - 26.2%] | 7.6% [6.8 - 8.6%]  10.4% [9.4 - 11.5%] |

Not sufficiently treated depressive subjects (anti-depressive medication [ATC-code N06A] and PHQ-9 ≥10), sufficiently treated depressive subjects (anti-depressive medication and PHQ-9 <10), depressive subjects without medication (no anti-depressive medication and PHQ-9 ≥10) and subjects with no anti-depressive medication and PHQ-9 <10)
